# Maternal dietary patterns and risk of gestational diabetes mellitus in twin pregnancies: a longitudinal twin pregnancies birth cohort study

**DOI:** 10.1186/s12937-020-00529-9

**Published:** 2020-02-10

**Authors:** Li Wen, Huisheng Ge, Juan Qiao, Lan Zhang, Xuyang Chen, Mark D. Kilby, Ying Zhou, Jie Gan, Richard Saffery, Jianying Yan, Chao Tong, Hongbo Qi, Philip N. Baker

**Affiliations:** 1 Department of Obstetrics, The First Affiliated Hospital of Chongqing Medical University, Chongqing, 400016 China; 2 International Collaborative Laboratory of Reproduction and Development, Ministry of Education, Chongqing Medical University, Chongqing, 400016 China; 3 State Key Laboratory of Maternal and Fetal Medicine of Chongqing Municipality, The First Affiliated Hospital of Chongqing Medical University, Chongqing, 400016 China; 4 Chengdu Women’s and Children’s Central Hospital, School of Medicine, University of Electronic Science and Technology of China, Chengdu, 611731 China; 5 Fetal Medicine Centre, Birmingham Women’s & Children’s Foundation Trust, Birmingham, B15 2TG UK; 6 Institute of Metabolism & Systems Research, University of Birmingham, Birmingham, B15 2TT UK; 7 Cancer, Disease and Developmental Epigenetics, Murdoch Children’s Research Institute, and Department of Paediatrics University of Melbourne, Parkville, Victoria 3052 Australia; 8 Fujian Provincial Maternity and Children’s Hospital, Affiliated Hospital of Fujian Medical University, Fuzhou, 350001 Fujian China; 9 College of Life Sciences, University of Leicester, Leicester, LE1 7RH UK

**Keywords:** Dietary patterns, Gestational diabetes, Twin pregnancies, China

## Abstract

**Background:**

Gestational diabetes mellitus (GDM) is correlated with an increased risk of adverse perinatal outcomes for both the mother and offspring. Previous research has reported correlations between maternal dietary patterns and GDM, but such evidence for twin pregnancies is lacking. This study aimed to identify maternal dietary patterns in the second trimester and investigate their relationships with the risk of GDM among women who were pregnant with twins in China.

**Methods:**

A longitudinal twin pregnancies birth cohort study of women who were pregnant with twins in China was conducted. Maternal dietary intake in the second trimester was recorded by using a food frequency questionnaire prior to the diagnosis of GDM among participants from the prospective twin pregnancies birth cohort in Chongqing City. GDM was diagnosed with a 75 g 2-h oral glucose tolerance test at 23–26 weeks of gestation. Dietary patterns were identified by principal components analysis, and the correlations between dietary pattern and GDM were examined using multivariable logistic regression analyses.

**Results:**

Of the 324 participants, 101 (31.2%) were diagnosed with GDM. Four dietary patterns were identified: a vegetable-based pattern, a poultry-and-fruit-based pattern, a sweet-based pattern and a plant-protein-based pattern. Multivariate analysis showed that none of the dietary patterns were correlated with the risk of GDM among women who were pregnant with twins, but the sweet-based dietary pattern, which was associated with a higher GDM risk for quartile 4 versus quartile 1 (OR 2.69; 95% CI: 1.09, 6.66) among non-overweight women (prepregnancy BMI < 24.0).

**Conclusion:**

Dietary patterns were not correlated with later GDM risk among women who were pregnant with twins in western China, whereas a high intake of sweets was associated with a higher risk for GDM among women who were not overweight prior to pregnancy.

**Trial registration:**

ChiCTR-OOC-16008203. Retrospectively registered on 1 April 2016.

## Introduction

Gestational diabetes mellitus (GDM) is one of the most common pregnancy complications in which women present with impaired glucose tolerance with an onset or first recognition during pregnancy [1, 2]. The prevalence of GDM varies from 9.8 to 25.5% according to the latest diagnostic criteria established by the International Association of Diabetes and Pregnancy Study Groups (IADPSG) in 2010 [3]. A previous study based on a large population suggested that the morbidity rate of GDM in the Chinese population varied from 17.5 to 18.9% according to the IADPSG criteria [4]. GDM is correlated with adverse effects on mothers and their offspring, such as macrosomia and cesarean section [5]. Although the blood glucose level of GDM patients usually returns to normal within 6 weeks after delivery, GDM increases the risk of postpartum type 2 diabetes among mothers and the risks of obesity or other metabolic complications among the offspring in their later life [6].

Given the known and potential adverse effects of GDM, the identification of risk factors for GDM is warranted. Accumulating evidence has revealed that dietary intake during pregnancy is involved in the development of GDM. High consumption of saturated fat, carbohydrates or animal protein is associated with a higher risk of GDM [7–10], while polyunsaturated fat intake appears to lower GDM risk [11], but debate on this topic remains [12]. To determine the correlation between food intake and perinatal outcomes, dietary pattern analyses are preferred with the advantages of accounting for food consumption over a given period and taking into account nutrients consumed in combination. For example, a systematic review suggested that vegetarian- or Mediterranean-style dietary patterns reduce the risk of GDM [13], whereas dietary patterns characterized by high intakes of red and processed meat and refined grains are associated with an increased risk of GDM [14, 15].

An increasing proportion of twin pregnancies has been observed worldwide in recent decades. Since women pregnant with twins are believed to undergo more complicated physiological changes and have a higher risk of adverse obstetric outcomes when compared to those with singleton pregnancies [16], it is essential to pay close attention to maternal and fetal health in the context of twin pregnancies. Previous studies have reported that twin gestation is associated with an increased risk of GDM [17, 18], and GDM was associated with a higher risk of gestational hypertension and preeclampsia in twin pregnancies [19]. Therefore, the influence of dietary intake on GDM in the context of twin pregnancies is worth exploring.

To date, studies on the effects of dietary habits on GDM development in the context of twin pregnancies are extremely limited. Therefore, the objective of this study is to identify maternal dietary patterns during pregnancy and investigate whether maternal dietary patterns are associated with the risk of developing GDM in the context of twin pregnancies in a Chinese prospective cohort.

## Methods

### Study design and participants

The current study was conducted with women pregnant with twins in the Chongqing Longitudinal Twin Study (LoTiS) (ChiCTR-OOC-16008203)—the world’s largest prospective twin pregnancies birth cohort that was established in Chongqing, China in 2016 with the main aim of elucidating the complex interplay between early-life environmental and genetic risk factors in the context of health and diseases [20]. Study participants were recruited at 11–16 weeks’ gestation from the First Affiliated Hospital of Chongqing Medical University and Chongqing Women and Children’s Health Center between January 2016 and September 2018. Four follow-up clinic visits were carried out throughout pregnancy, and eight pediatric follow-up visits were conducted 3 years after birth. The LoTiS study was approved by the Ethics Committee of the First Affiliated Hospital of Chongqing Medical University (No.201530). Written informed consent was obtained from all participants. Participants were subjected to a 75 g oral glucose tolerance test (OGTT) between the 23rd and 26th weeks of gestation, and those who completed a food frequency questionnaire prior to the diagnosis of GDM on the same day were eligible for this study.

### Dietary assessment

The maternal average dietary intake over the past 3 months was assessed using a food frequency questionnaire (FFQ) and was recorded correctly by a trained researcher in a face-to-face interview. The FFQ originated from Singapore and has been validated in a singleton pregnancy study conducted in our laboratory [21]. The FFQ consists of 93 specified food items as well as 15 additional questions about dietary behaviors. The participants were asked to recall food intake frequencies (how many times per day or per week or per month) and estimate the food intake portion each time each food item listed was consumed. A photo booklet was shown to participants to help them understand the standard portion sizes. The quantities and frequencies were recorded in detail. The dietary information of individuals was entered electronically for further calculation.

We calculated the daily food intake by averaging the consumption frequency of each food item per day and adjusting the daily food intake for energy intake based on the China Food Composition Database. Some food items were combined into one group of items with similar nutrient profiles or culinary uses. Forty non-overlapping food groups served as the main dataset for investigation. The total frequency of the intake of items in one food group was the sum of all food items consumed in the group.

### Diagnosis of gestational diabetes mellitus

GDM was diagnosed by a 75 g 2-h OGTT only when the following plasma glucose values were met or exceeded according to the IADPSG (International Association of et al., 2010): fasting glucose ≥5.1 mmol/L, 1 h glucose ≥10.0 mmol/L, or 2 h glucose ≥8.5 mmol/L.

### Perinatal outcomes

Perinatal outcomes other than GDM were obtained from medical records, including gestational hypertension (GHT), preeclampsia (PE), hypothyroidism (HT), intrahepatic cholestasis of pregnancy (ICP), spontaneous preterm birth (sPTB). In addition, the information of gestational age at delivery, delivery mode, birth weight and NICU admission were also collected.

### Covariates

Covariates were assessed using a structured questionnaire at the recruitment interview. We collected data on maternal age, ethnicity (Han Chinese, others), education level (junior secondary school or below, senior/technical secondary school, university or above), smoking status before pregnancy, parity (0, ≥1), chorionicity (monochorionic-diamniotic, dichorionic-diamniotic), mode of conception (naturally conceived, in vitro fertilization-embryo transfer), previous history of GDM, family history of GDM (first-degree relatives). Prepregnancy BMI (kg/m^2^) was calculated as the ratio of weight (kg) to squared height (m^2^), which was calculated from self-reported prepregnancy weight and measured height.

### Statistical analyses

Principal component analysis with orthogonal (varimax) rotation was used to derive dietary patterns. We standardized the consumption frequency of each food group according to the mean and standard deviation before the extraction of dietary patterns. Four dietary patterns formed by linear combinations of each food group were selected by an inspection of scree plots and the interpretability of the results. The factor loadings, also known as coefficients defining these linear combinations, reflect the correlations of food groups with the corresponding dietary pattern. Food groups with loadings > 0.2 were used to describe each dietary pattern. We calculated factor scores for each dietary pattern by summing the consumption frequencies of each food group and multiplying the sum by the factor loadings for each participant, and we categorized participants into quartiles based on their dietary pattern scores for subsequent analyses.

Frequencies and percentages are used to describe the distributions of categorical variables and continuous variables are expressed as the means ± SD. Chi-square tests or Fisher’s exact tests were used to compare categorical variables between groups, and the continuous variables among groups were compared by using student t test. The logistic regression models were used to estimate the odds ratio (OR) and 95% confidence interval (CI) for GDM related to dietary pattern quartiles. We used the lowest quartile of the dietary pattern score as a reference. Multivariate linear regression models were used to examine the association between dietary pattern scores and plasma glucose levels following the OGTT. We conducted crude and adjusted analyses using the following models: Model 1, the crude model (individual dietary pattern); Model 2, Model 1 plus other dietary patterns; and Model 3, Model 2 plus maternal age, ethnicity, prepregnancy BMI, education level, smoking status, parity, previous history of GDM and family history of diabetes mellitus (DM). We also examined potential effect modification by age and prepregnancy weight status by including multiplicative interaction terms in the models.

All analyses were performed with SPSS software version 22.0 (SPSS, Inc.). For all statistical analyses, a two-tailed *p*-value less than 0.05 was considered statistically significant.

## Results

### Characteristics of the participants

**Fig. 1:**
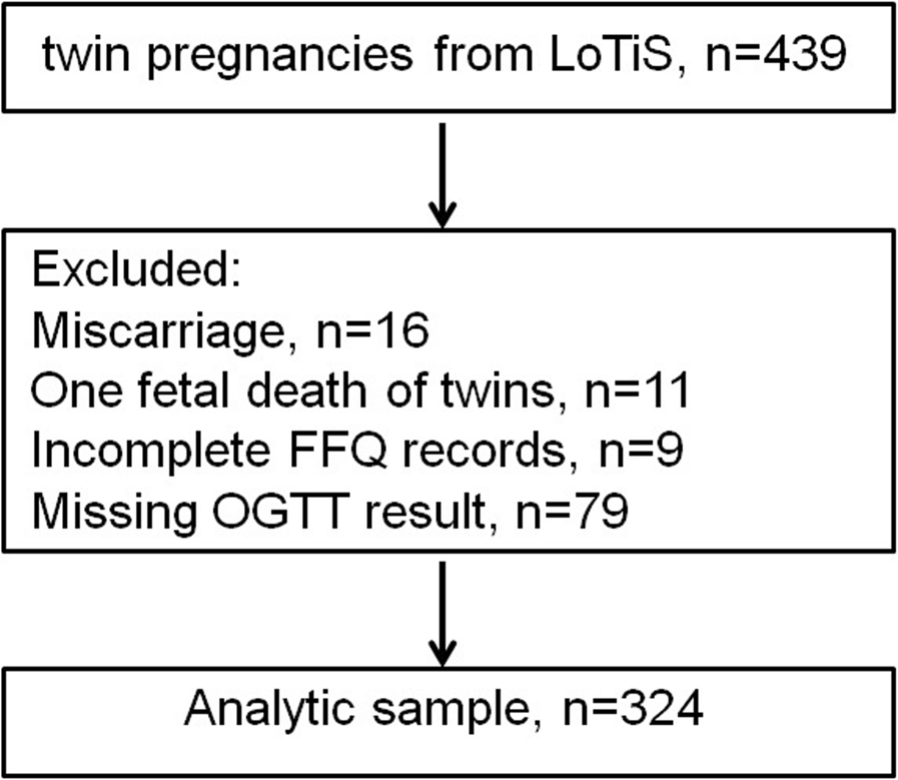
Flowchart showing selection of participants included in this analysis from LoTiS study

After excluding women pregnant with twins who had a miscarriage (*n* = 16), who experienced fetal death of one of the twins (*n* = 11), who had incomplete FFQ records (*n* = 9), and who had missing OGTT results (*n* = 79), a total of 324 women were available for analysis (Fig. 1). There were no significant differences in regard to age, ethnicity, prepregnancy BMI, mode of conception, chorionicity or parity between the women who were included and those who were excluded.

The incidence of GDM was 31.2% in this study population (101 out of 324 pregnant women). Table 1 summarizes the participant characteristics according to GDM status. Overall, no significant differences were observed between GDM and non-GDM women in terms of ethnicity, education level, smoking status before pregnancy, chorionity, parity, mode of conception, previous history of GDM, family history of T2DM and energy intake. However, compared to women without GDM, women with GDM tended to be older (≥ 35 years old) and were more likely to have a BMI higher than 24.0 kg/m^2^ before pregnancy (26.7% versus 17.5%) (*p* < 0.01 for both).

**Table 1:** Characteristics of participants by GDM status^a^

Variables	Overall (*n* = 324)	GDM (*n* = 101)	Non-GDM (*n* = 223)	*P*-value^b^
Maternal age (y)				0.001
< 25	36 (11.1)	3 (3.0)	33 (14.8)	
25–29	137 (42.3)	49 (48.5)	88 (39.5)	
30–34	113 (34.9)	30 (29.7)	83 (37.2)	
≥ 35	38 (11.7)	19 (18.8)	19 (8.5)	
Pre-pregnancy BMI (kg/m^2^)				0.001
< 18.5	39 (12.0)	3 (3.0)	36 (16.1)	
18.5–23.9	219 (67.6)	71 (70.3)	148 (66.4)	
≥ 24.0	66 (20.4)	27 (26.7)	39 (17.5)	
Ethnicity				0.743
Han Chinese	303 (93.5)	97 (96.0)	206 (92.4)	
Others	21 (6.5)	4 (4.0)	17 (7.6)	
Education level				0.486
Junior secondary school or below	52 (16.0)	14 (13.9)	38 (17.0)	
Senior/technical secondary school	36 (11.1)	14 (13.9)	22 (9.9)	
University or above	236 (72.8)	73 (72.3)	163 (73.1)	
Smoking before pregnancy				0.872
No	307 (94.8)	96 (95.0)	211 (94.65)	
Yes	17 (5.2)	5 (5.0)	12 (5.4)	
Mode of conception				0.122
Natural conceived	194 (59.9)	51 (55.0)	143 (64.1)	
In vitro fertilization-embryo transfer	130 (40.1)	50 (45.0)	80 (35.9)	
Chorionicity				0.815
Monochorionic-diamniotic	136 (42.0)	40 (45.0)	96 (43.0)	
Dichorionic-diamniotic	188 (58.0)	61 (55.0)	127 (57.0)	
Parity				0.586
0	239 (73.8)	77 (76.2)	162 (72.6)	
≥ 1	85 (26.2)	24 (23.8)	61 (27.4)	
Previous history of GDM				1.000
No	319 (98.5)	99 (98.0)	220 (98.7)	
Yes	5 (1.5)	2 (2.0)	3 (1.3)	
Family history of T2DM				0.493
No	309 (95.4)	98 (97.0)	211 (94.6)	
Yes	15 (4.6)	3 (3.0)	12 (5.4)	

*BMI* Body mass index, *GDM* Gestational diabetes mellitus, *DM* Diabetes mellitus

^a^Numbers are presented as n (%)

^b^Based on χ^2^ tests

### Dietary pattern analysis

In the present study, four main maternal dietary patterns accounted for 28.44% of the total variation. The factor loading for each dietary pattern are listed in Table 2. The first pattern, named the “vegetable-based pattern”, explained 9.24% of the total variance. This pattern was characterized by a high intake of root vegetables, gourd/melon family vegetables, freshwater fish, leafy and cruciferous vegetables, and red meat. The second pattern, named the “poultry-and-fruit-based pattern”, explained 7.40% of the total variance. This pattern was characterized by a high intake of poultry, fresh fruit, processed fruit, soups and meat innards. The third pattern, named the “sweet-based pattern”, explained 5.97% of the total variance. This pattern was characterized by a high intake of biscuits, pastries, cakes, bread and deep-sea fish and seafood products. The fourth pattern, named the “plant-protein-based pattern”, explained 5.83% of the total variance. This pattern was characterized by soya milk, legumes, beans or bean products, buns and rice.

**Table 2:** Factor loadings for the four dietary patterns identified from principal components analysis^a^

Dietary pattern	Food	Factor loading coefficient	Cumulative variance explained
Vegetable-based pattern	Root vegetables	0.723	9.24
	Gourd/melon family vegetables	0.686	
	Freshwater fish	0.491	
	Leafy and cruciferous vegetables	0.488	
	Red meat	0.471	
	Fried breads	0.370	
	White rice	0.301	
	Deep-sea fish and seafood products	0.268	
	Dumplings	−0.222	
Poultry-fruit-based pattern	Poultry	0.544	16.64
	Fresh fruit	0.505	
	Processed fruit	0.447	
	Soups	0.424	
	Meat Innards	0.422	
	Noodles	0.373	
	Cereals	0.347	
	Dairy products	0.346	
	Deep-sea fish and seafood products	0.322	
	Nuts/seeds	0.319	
	Eggs	0.315	
	White rice	−0.267	
Sweets -based pattern	Biscuits\pastries\cakes	0.560	22.61
	Bread	0.455	
	Deep-sea fish and seafood products	0.314	
	Ethnic breads	0.313	
	Dessert soup	0.307	
	Dairy products	0.296	
	Noodles	−0.436	
	Meat products	−0.405	
	Dumplings	−0.267	
Plant protein-rich--based pattern	Soya milk	0.670	28.44
	Legumes	0.644	
	Beans/bean products	0.535	
	Bun	0.442	
	Rice	0.421	
	Bread	0.407	
	Ethnic breads	0.236	
	Cereals	−0.306	

^a^Factor loading < ±0.200 were not listed in the table for simplicity

Table 3 describes the participants’ characteristics and their dairy energy consumption according to the quartiles of dietary pattern scores. Regarding the vegetable-based pattern, women with the highest score tended to be highly educated, were more likely to have monochorionic-diamniotic twin pregnancies and had higher intakes of total energy than those with the lowest score. For the poultry-and-fruit-based pattern, women with the highest score were more likely to have a IVF-ET mode of conception, and more likely to have dichorionic-diamniotic twin pregnancies than those with the lowest score. For the sweet-based pattern, women with the highest score had higher intakes of total energy and tended to have a higher incidence of GDM, but the GDM incidence was not significantly higher than that of those with the lowest score. Regarding the plant-protein-based pattern, women with the highest score had higher intakes of total energy than those with the lowest score.

**Table 3:** Characteristics and dairy energy consumption of participants by quartiles of dietary pattern scores^a^

Variables	Vegetable		Poultry-fruit		Sweets		Plant protein-rich	
	Q1	Q4	Q1	Q4	Q1	Q4	Q1	Q4
GDM								
No	59(72.8)	60(74.1)	58(71.6)	54(66.7)	61(75.3)	49(60.5)	55(67.9)	54(66.7)
Yes	22(27.2)	21(25.9)	23(28.4)	27(33.3)	20(24.7)	32(39.5)	26(32.1)	27(33.3)
*P*^b^	1.000		0.610		0.064		1.000	
Maternal age (y)								
< 25	9(11.1)	6(7.4)	10(12.3)	7(8.6)	9(11.1)	9(11.1)	13(16.1)	5(6.2)
25–29	27(33.3)	33(40.7)	35(43.2)	32(39.5)	31(38.3)	37(45.7)	35(43.2)	33(40.7)
30–34	37(45.7)	32(39.6)	28(34.6)	34(42.0)	35(43.2)	26(32.1)	26(32.1)	35(43.2)
≥ 35	8(9.9)	10(12.3)	8(9.9)	8(9.9)	6(7.4)	9(11.1)	7(8.6)	8(9.9)
*P*^b^	0.634		0.757		0.491		0.172	
Pre-pregnancy BMI (kg/m^2^)								
< 18.5	6(7.4)	13(16)	10(12.3)	11(13.6)	9(11.1)	9(11.1)	12(14.8)	10(12.3)
18.5–23.9	58(71.6)	55(68)	52(64.2)	56(69.1)	56(69.1)	59(72.8)	53(65.4)	54(66.7)
≥ 24.0	17(21)	13(16)	19(23.5)	14(19.8)	16(19.8)	13(16.1)	16(19.8)	17(21)
*P*	0.206		0.664		0.825		0.944	
Ethnicity								
Han Chinese	72(88.9)	79(97.5)	79(97.5)	76(93.8)	74(91.4)	75(92.6)	72(88.9)	79(97.5)
Others	9(11.1)	2(2.5)	2(2.5)	5(6.2)	7(8.6)	6(7.4)	9(11.1)	2(2.5)
*P*^b^	0.057		0.443		1.000		0.057	
Education level								
Junior secondary school or below	13(16)	11(13.6)	15(18.5)	11(13.6)	15(18.5)	8(9.9)	16(19.8)	13(16)
Senior/technical secondary school	12(14.8)	3(3.7)	7(8.6)	12(14.8)	12(14.8)	7(8.6)	10(12.3)	7(8.7)
University or above	56(69.1)	67(82.7)	59(72.9)	58(71.6)	54(66.7)	66(81.5)	55(67.9)	61(75.3)
*P*^b^	0.035		0.346		0.113		0.587	
Smoking before pregnancy								
No	75(92.6)	76(93.8)	77(95.1)	77(95.1)	79(97.5)	77(95.1)	76(93.8)	78(96.3)
Yes	6(7.4)	5(6.2)	4(4.9)	4(4.9)	2(2.5)	4(4.9)	5(6.2)	3(3.7)
*P*^b^	1.000		1.000		0.682		0.720	
Mode of conception								
Natural conceived	43(53.1)	54(66.7)	62(76.5)	39(48.1)	50(61.7)	40(49.4)	44(54.3)	44(54.3)
IVF-ET	38(46.9)	27(33.3)	19(23.5)	42(51.9)	31(38.3)	41(50.6)	37(45.7)	37(45.7)
*P*^b^	0.109		**< 0.001**		0.155		1.000	
Chorionicity								
Monochorionic-diamniotic	23(28.4)	40(49.4)	40(49.4)	27(33.3)	33(40.7)	33(40.7)	33(40.7)	31(38.3)
Dichorionic-diamniotic	58(71.6)	41(50.6)	41(50.6)	54(66.7)	48(59.3)	48(59.3)	48(59.3)	50(61.7)
*P*^b^	0.010		0.055		1.000		0.872	
Parity								
0	58(71.6)	60(74.1)	58(71.6)	65(80.2)	59(72.8)	67(82.7)	61(75.3)	61(75.3)
≥ 1	23(28.4)	21(25.9)	23(28.4)	16(19.8)	22(27.2)	14(17.3)	20(24.7)	20(24.7)
*P*^b^	0.860		0.270		0.185		1.000	
Previous history of GDM								
No	81(100)	80(98.8)	80(98.8)	79(97.5)	80(98.8)	79(97.5)	81(100)	79(97.5)
Yes	0(0)	1(1.2)	1(1.2)	2(2.5)	1(1.2)	2(2.5)	0(0)	2(2.5)
*P*^b^	1.000		1.000		1.000		1.000	
Family history of DM								
No	78(96.3)	77(95.1)	79(97.5)	78(96.3)	76(93.8)	79(97.5)	77(95.1)	76(93.8)
Yes	3(3.7)	4(4.9)	2(2.5)	3(3.7)	5(6.2)	2(2.5)	4(4.9)	5(6.2)
*P*^b^	1.000		1.000		0.443		1.000	
Energy intake, kcal/d								
≥ 2100	17(21)	44(54.3)	12(14.8)	21(25.9)	25(30.9)	45(55.6)	27(33.3)	47(58)
< 2100	64(79)	37(45.7)	69(85.2)	60(74.1)	56(69.1)	36(44.4)	54(66.7)	34(42)
*P*^b^	< 0.001		0.118		0.002		0.003	

*BMI* Body mass index, *GDM* Gestational diabetes mellitus, *DM* Diabetes mellitus

^a^ Numbers are presented as n (%)

^b^ Based on χ^2^ tests

Besides, the analyses of perinatal outcomes other than GDM according to the quartiles of dietary pattern scores was shown in Table 4. There were no correlations found between dietary patterns and the other pregnancy outcomes, except birth weight. The larger co-twin birth weight of women with the highest score in vegetable-based pattern is significantly lower than that of women with the lowest score in vegetable-based pattern. Similar trend has also been observed in the smaller co-twin, although statistical significance wasn’t achieved. These facts indicate that women with vegetable-based pattern during the second trimester are more likely to deliver lighter offspring.

**Table 4:** Comparison of other pregnancy outcomes between quartiles of dietary pattern scores^a^

Variables	Vegetable		Poultry-fruit		Sweets		Plant protein-rich	
	Q1	Q4	Q1	Q4	Q1	Q4	Q1	Q4
GHP								
No	78(96.3)	75(92.6)	80(98.8)	77(95.1)	79(97.5)	75(92.6)	78(96.3)	78(96.3)
Yes	3(3.7)	6(7.4)	1(1.2)	4(4.9)	2(2.5)	6(7.4)	3(3.7)	3(3.7)
*P*^b^	0.495		0.367		0.277		1.000	
PE								
No	77(95.1)	74(91.4)	77(95.1)	76(93.8)	77(95.1)	76(93.8)	79(97.5)	75(92.6)
Yes	4(4.9)	7(8.6)	4(4.9)	5(6.2)	4(4.9)	5(6.2)	2(2.5)	6(7.4)
*P*^b^	0.540		1.000		1.000		0.277	
HT								
No	79(97.5)	78(96.3)	77(95.1)	78(96.3)	78(96.3)	77(95.1)	76(93.8)	77(95.1)
Yes	2(2.5)	3(3.7)	4(4.9)	3(3.7)	3(3.7)	4(4.9)	5(6.2)	4(4.9)
*P*^b^	1.000		1.000		1.000		1.000	
ICP								
No	77(95.1)	70(86.4)	72(88.9)	70(86.4)	70(86.4)	71(87.7)	69(85.2)	71(87.7)
Yes	4(4.9)	11(13.6)	9(11.1)	11(13.6)	11(13.6)	10(12.3)	12(14.8)	10(12.3)
*P*^b^	0.058		0.812		1.000		0.819	
GA (wk)	36.6 ± 1.5	36.3 ± 1.6	36.5 ± 1.5	36.3 ± 1.6	36.4 ± 1.7	36.3 ± 1.4	36.3 ± 1.6	36.6 ± 1.1
*P*^c^	0.242		0.399		0.772		0.132	
sPTB								
No	60(74.1)	63(77.8)	66(81.5)	62(76.5)	65(80.2)	55(67.9)	64(79.0)	64(79.0)
Yes	21(25.9)	18(22.2)	15(18.5)	19(23.5)	16(19.8)	26(32.1)	17(21.0)	17(21.0)
*P*^b^	0.714		0.563		0.106		1.000	
Cesarean								
No	0(0.0)	1(1.2)	2(2.5)	1(1.2)	2(2.5)	0(0.0)	0(0.0)	0(0.0)
Yes	81(100)	80(98.8)	79(97.5)	80(98.8)	79(97.5)	81(100)	81(100)	81(100)
*P*^b^	1.000		1.000		0.497		1.000	
BW^H^ (g)	2675 ± 424	2525 ± 408	2628 ± 413	2649 ± 408	2585 ± 424	2637 ± 360	2602 ± 425	2690 ± 388
*P*^c^	0.024		0.750		0.394		0.174	
BW^L^ (g)	2389 ± 434	2262 ± 412	2354 ± 391	2368 ± 411	2311 ± 431	2380 ± 392	2323 ± 456	2383 ± 401
*P*^c^	0.059		0.827		0.284		0.377	
NICU								
No	68(84.0)	61(75.3)	63(77.8)	65(80.2)	65(80.2)	63(77.8)	66(81.5)	68(84.0)
Yes	13(16.0)	20(24.7)	18(22.2)	16(19.8)	16(19.8)	18(22.2)	15(18.5)	13(16.0)
*P*^b^	0.242		0.847		0.847		0.836	

*GHP* Gestational hypertension, *PE* Preeclampsia, *HT* Hypothyroidism, *ICP* Intrahepatic cholestasis of pregnancy, *GA* Gestational age, *sPTB* Spontaneous preterm birth, *BW*^*H*^ Heavier twin birthweight, *BW*^*L*^ Lighter twin birthweight

^a^ Numbers are presented as n (%) or means±SD

^b^ Based on χ^2^ tests or Fisher exact test

^c^ Based on student t test

### Dietary patterns and risk of gestational diabetes mellitus

Table 5 summarizes the univariate and multivariate regression analyses for the correlation between dietary pattern and risk of GDM. There was no significant correlation between any dietary pattern and the risk of GDM. Compared with the lowest quartiles of the dietary pattern scores, the multivariable-adjusted ORs for the corresponding highest quartile of the vegetable-based, poultry-and-fruit-based, sweet-based and plant-protein-based patterns were 1.23 (95% CI: 0.57, 2.66, *p* > 0.05), 0.96 (95% CI: 0.45, 2.03, *p* > 0.05), 1.97 (95% CI: 0.94, 4.12, *p* > 0.05) and 1.02 (95% CI: 0.49, 2.09, *p* > 0.05), respectively.

**Table 5:** Logistic regression analysis for the risk of GDM according to the quartiles of dietary pattern scores

Dietary patterns	Q1 (*n* = 81)	Q2 (*n* = 81)	Q3 (*n* = 81)	Q4 (*n* = 81)	*P* for trend
	Reference	Odds Ratio (OR) (95% CI)			
Vegetable-based pattern					
Model 1^a^	1.00	1.20 (0.61–2.36)	1.14 (0.65–2.57)	0.94 (0.47–1.89)	0.169
Model 2^b^	1.00	1.18 (0.59–2.36)	1.12 (0.61–2.24)	0.98 (0.47–2.01)	0.235
Model 3^c^	1.00	1.50 (0.72–3.14)	1.89 (0.90–3.98)	1.23 (0.57–2.66)	0.357
Poultry-fruit-based pattern					
Model 1^a^	1.00	1.12 (0.58–2.19)	1.00 (0.51–1.96)	1.19 (0.60–2.31)	0.943
Model 2^b^	1.00	1.10 (0.55–2.18)	1.05 (0.51–2.16)	1.10 (0.54–2.20)	0.994
Model 3^c^	1.00	1.02 (0.50–2.11)	0.98 (0.46–2.10)	0.96 (0.45–2.03)	0.999
Sweets-based pattern					
Model 1^a^	1.00	1.36 (0.68–2.72)	1.28 (0.64–2.57)	1.99 (1.02–3.91)	0.236
Model 2^b^	1.00	1.24 (0.61–2.52)	1.17 (0.57–2.38)	1.84 (0.92–3.69)	0.340
Model 3^c^	1.00	1.37 (0.65–2.89)	1.34 (0.64–2.83)	1.97 (0.94–4.12)	0.349
Plant protein-rich-based pattern					
Model 1^a^	1.00	1.18 (0.61–2.28)	0.78 (0.40–1.56)	0.82 (0.58–2.07)	0.649
Model 2^b^	1.00	1.13 (0.58–2.22)	0.75 (0.37–1.54)	0.81 (0.59–1.92)	0.655
Model 3^c^	1.00	0.98 (0.48–2.00)	0.68 (0.32–1.43)	1.02 (0.49–2.09)	0.667

^a^Crude model

^b^Adjusted for other dietary patterns

^c^Model 2 plus maternal age, pre-pregnancy BMI, ethnicity, education level, parity, smoking status, chorionicity, mode of conception, previous history of GDM and family history of DM

**Fig. 2:**
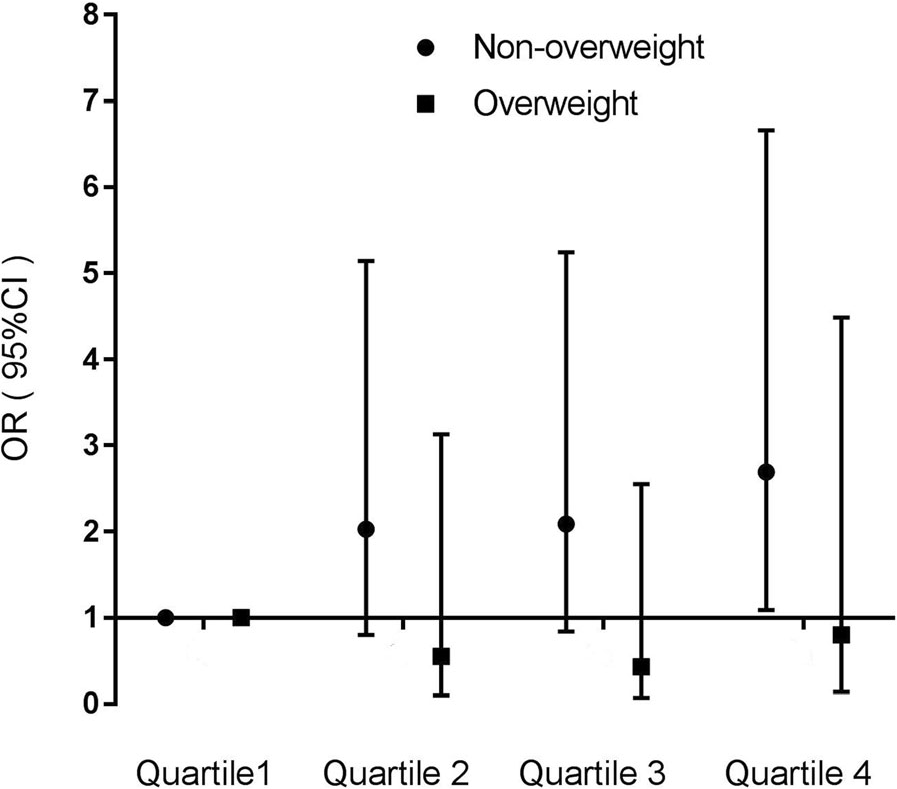
Associations between sweets-based pattern score quartiles and the risk of gestational diabetes mellitus, stratified by pre-pregnancy body mass index levels (< 24.0 vs. ≥ 24.0). Adjusted for other dietary pattern, maternal age, ethnicity, education level, parity, smoking status, chorionicity, mode of conception, previous history of GDM and family history of DM. (●) represents women with BMI < 24.0 kg/m^2^; (■) represents women with BMI ≥ 24.0 kg/m^2^

In the subgroup analyses (Fig. 2), a significant increase in GDM risk was observed only among nonoverweight women (prepregnancy BMI < 24.0) when comparing the highest quartile of sweet-based pattern scores to the lowest quartile (OR 2.69; 95% CI: 1.09, 6.66; *p* < 0.05), despite the lack of significance for the interaction between prepregnancy BMI and sweets-based pattern score (p for interaction =0.267). There was no effect modification by prepregnancy BMI on the association between other dietary patterns and GDM risk. There were no modification effects of any dietary patterns by age.

Furthermore, we examined the association of the sweet-based pattern with blood glucose levels following the OGTT, and the results showed that the sweet-based pattern was positively correlated with 1 h postload blood glucose among nonoverweight women (β 0.18; 95% CI: 0.01, 0.35; *p* < 0.05) (Table 6).

**Table 6:** Linear regression analysis of correlations between the sweet-based pattern and blood glucose levels following OGTT

Variables	Blood Glucose Level β (95% CI)					
	Fasting	*p*-value	1 h after OGTT	*p*-value	2 h after OGTT	*p*-value
Non-overweight						
Model 1^a^	0.03 (−0.0–0.07)	0.117	0.20 (0.03–0.37)	0.020	0.12 (−0.02–0.26)	0.091
Model 2^b^	0.03 (−0.01–0.07)	0.103	0.18 (0.01–0.36)	0.039	0.11 (−0.04–0.25)	0.148
Model 3^c^	0.03 (−0.01, 0.07)	0.102	0.18 (0.01–0.35)	0.039	0.12 (−0.02–0.26)	0.103
Overweight						
Model 1^a^	−0.07 (− 0.17–0.03)	0.181	−0.24 (− 0.63–0.15)	0.228	−0.18 (− 0.48–0.13)	0.250
Model 2^b^	− 0.08 (− 0.18–0.03)	0.145	−0.24 (− 0.62–0.15)	0.218	−0.17 (− 0.47–0.14)	0.287
Model 3^c^	− 0.08 (− 0.17–0.02)	0.133	−0.23 (− 0.61–0.16)	0.248	−0.17 (− 0.48–0.14)	0.275

^a^Crude model

^b^Adjusted for other dietary patterns

^c^Model 2 plus maternal age, ethnicity, education level, parity, smoking status, chorionicity, mode of conception, previous history of GDM and family history of DM

## Discussion

In this Chinese prospective twin pregnancies birth cohort study, four dietary patterns, namely, the vegetable-based pattern, the poultry-and-fruit-based pattern, the sweet-based pattern and the plant-protein-based pattern, were identified in the second trimester. No significant association was found between the four dietary patterns and the risk of GDM. However, we observed that the sweet-based pattern was significantly associated with an increased risk of GDM and higher blood glucose levels 1 h after the OGTT only among nonoverweight women. It is speculated that dietary intake might have little influence on prepregnancy nonoverweight women but not on prepregnancy overweight women.

In recent years, the impact of food intake on the risk of GDM has gained increasing attention. Dietary pattern analysis is a holistic approach to account for food consumption in a typical diet and take the synergy of food and nutrient intake into account. The majority of studies on dietary patterns and GDM risk were first conducted in Western populations. Generally, these studies found that a prudent dietary pattern that was high in seafood, eggs, vegetables, fruits, berries and vegetable oils [22], a prudent diet that was high in fruits, green vegetables and fish [23], and a ‘Mediterranean’ dietary pattern [24] were associated with a lower risk of GDM, while a ‘Western’ dietary pattern that was high in red and processed meat, French fries, pizza, sweets and desserts was positively associated with a higher risk of GDM [23]. Since the heterogeneity of dietary structure among the different countries or regions results in different dietary patterns, we paid special attention to studies that were conducted in the Chinese population [25–29]. In general, a western pattern high in dairy products and baked/fried food, and a sweet pattern high in cantonese desserts and sugar-sweetened beverages were related to an increased risk of GDM, which were similar with the findings in a Western population [30], whereas a vegetable pattern rich in root vegetables, beans and melon vegetables was related with a decreased risk of GDM. Due to the diversity of dietary traditions across China, the definition of ‘Chinese traditional’ diet pattern was not uniformed, and therefore, results in conflicting conclusions. For instance, a ‘traditional pattern’ that was high in vegetables, fruits, and rice was associated with a decreased risk of GDM [27, 29], whereas a ‘traditional pattern’ defined as the high intake of vegetables, fine grains, red meat and tubers was associated with an increased risk of GDM [26], the researchers in this study suggested that the increased likelihood of GDM may result from the effect of red meat.

Unlike prior research in the context of singleton pregnancies, the present study found no significant correlations between the identified dietary patterns and the risk of GDM in women pregnant with twins. Since previous studies have reported that associations between dietary patterns and GDM may vary by maternal characteristics, such as maternal age [29], prepregnancy BMI [22, 28], and maternal family history of diabetes [25], we examined potential effect modification by age and prepregnancy weight status. A significant association was observed between high sweet food intake and the risk of GDM among non-overweight women, whereas no association was found among overweight women. Additionally, we found that high sweet food intake influenced 1-h blood glucose levels following the OGTT among nonoverweight women. There were no modification effects of any dietary patterns by age. One possible reason may be due to the high incidence of GDM in this study (31.2%). The environmental exposure factors had limited influence on the blood glucose levels, which was consistent with our previous result showing that there was no correlation between gestational weight gain and the incidence of GDM [31]. An alternative explanation was that there may be differences between twin and singleton gestation in terms of the development of GDM. A study showed that the mean serum concentration of human placental lactogen (hPL) at 30 and 36 weeks of gestation was markedly elevated in twin pregnancies compared with the concentration in singleton pregnancies. Higher levels of hormones, such as hPL, estrogen and progesterone, in twin pregnancies may influence the frequency of GDM through their insulin antagonistic effects [32]. In addition, another study suggested that placental mass and the number of fetuses contribute to the occurrence of GDM [33]. These reports support the hypothesis that increasing placental mass and increasing diabetogenic hormones may play an important role in the etiology of GDM in twin pregnancies.

We did not detect an effect modification by maternal family history of diabetes since only 15 (4.6%) participants had a family history of T2DM. The rate of women with a family history of T2DM in our study appeared to be very low, but we could explain it. An epidemiological study of DM has shown that the prevalence of DM was 11.6–13.2% in Chongqing region, China [34]. On the other hand, a similar dietary pattern study performed in an adjacent area in western China revealed that 6.2% of pregnant women out of 1337 participants had a family history of T2D, and this rate is similar to that of our study [28]. Together with the relatively small sample size of this study, these factors lead to the low incidence of a family history of diabetes.

The strength of our study is the dietary patterns we identified reflected the habitual diet in the second trimester. Most pregnant women had a poor appetite or unusual tastes in the first trimester due to the gestational reactions of nausea and vomiting, and the dietary intake during this period has limited research significance. In the second trimester, pregnant women had a better appetite, and we used the FFQ to investigate their dietary intake, which has the advantage of capturing long-term habitual diet [35], additionally, GDM was diagnosed in this period. It is reasonable to explore the influence of dietary intake on the risk of GDM. Another strength of our study was the specific study population. This is the first study using a population pregnant with twins to explore the effects of dietary patterns on the risk of GDM.

This study contributes new knowledge regarding the relationship between dietary intake and risk of GDM in twin pregnancies, but several limitations of this study should be taken into consideration. Although it is a common sense that a prospective twin birth cohort is extremely difficult to establish, the relatively small sample size is a weakness of this study, as a sample size that is at least 5~10 fold the number of questionnaire items is required to attain adequate statistical power. Additionally, although FFQs have the advantage of capturing long-term habitual dietary intake, they have a limited ability to accurately and prospectively recording food intake [35]. Finally, the lack of information related to glycemic control after GDM was diagnosed could be improved in future studies. Blood glucose level assessment would be expected as a follow-up study to observe the short-term and long-term influences of GDM on maternal and neonatal outcomes. This study found no relationship between the vegetable dietary pattern during the second trimester and the incidence of GDM however, this dietary pattern may affect the birth weight of offspring in the presence or absence of GDM. However, the maternal nutrition status during the third trimester also has profound impact on fetal birth weight, further trimester-based nutritional investigations are warranted to decipher the correlation between vegetable dietary pattern and fetal birth weight of twin pregnancy.

## Conclusion

This is the first study to specifically investigate the effects of dietary patterns on the risk of GDM in a population pregnant with twins. Four dietary patterns were identified: a vegetable-based pattern, a poultry-and-fruit-based pattern, a sweet-based pattern and a plant-protein-based pattern. Although our study indicated that no dietary patterns were associated with the risk of GDM in twin pregnancies, there was a significant positive association between the sweet-based diet pattern characterized by a high intake of biscuits\pastries\cakes, breads, desserts and the incidence of GDM among women pregnant with twins who were not overweight prior to pregnancy. Further research is needed to elucidate the role of glucose levels in maternal and neonatal outcomes in Chinese women who are pregnant with twins.

## Data Availability

The datasets used and/or analyzed during the current study are available from the corresponding author upon request.

## Abbreviations

BMI: Body mass index
BW: Birth weight
CI: Confidence interval
FFQ: Food frequency questionnaire
GA: Gestational age
GDM: Gestational diabetes mellitus
GHT: Gestational hypertension
hPL: Human placental lactogen
HT: Hypothyroidism
IADPSG: International Association of Diabetes and Pregnancy Study Groups
ICP: Intrahepatic cholestasis of pregnancy
LoTiS: Longitudinal Twin Study
NICU: Neonatal Intensive Care Unit
OGTT: Oral glucose tolerance test
OR: Odds ratio
PE: Preeclampsia
sPTB: Spontaneous preterm birth
T2DM: Type 2 Diabetes Mellitus
